# Desmoid tumor of posterior cruciate ligament of the knee: a case report

**DOI:** 10.1186/1471-2474-14-69

**Published:** 2013-02-23

**Authors:** Wang Ling, Song Kedong, Wang Hong, Zhang Weiguo, Lv Decheng

**Affiliations:** 1 Department of Oncology, First Affiliated Hospital of Dalian Medical University, Dalian, People’s Republic of China; 2 Dalian R&D Center for Stem Cell and Tissue Engineering, Dalian University of Technology, Dalian 116024, China; 3 Department of Orthopaedics, First Affiliated Hospital of Dalian Medical University, Dalian, People’s Republic of China

**Keywords:** Desmoid tumor, Posterior cruciate ligament, Knee, Arthroscopical reconstruction, Surgery

## Abstract

**Background:**

Desmoid tumor is a rare type of cancer that develops in the tissues that form tendons and ligaments. These tumors, also called aggressive fibromatosis, are considered benign with no metastatic potential. They may invade nearby tissues and organs, however, and can be difficult to control. Desmoid tumor in the posterior cruciate ligament (PCL) of the knee has never been described in the literature.

**Case presentation:**

A 49-year-old man presented with a 2-month history of posteromedial knee dull pain and decreased range of motion of the knee. He was diagnosed desmoid tumor of posterior cruciate ligament of the knee by intraoperative biopsy, and underwent successful PCL resection and reconstruction by Four-strand semitendinosus and gracilis tendon autograft arthroscopically, and fortunately five years after operation, there were no clues as to recurrence of the tumor examined by Magnetic Resonance Imaging (MRI).

**Conclusion:**

Desmoid tumor is characterized by infiltrative growth and a tendency towards recurrence,as this tumor entity is rare, data giving evidence based recommendations for the optimal treatment algorithm for this disease is lacking. At present there is no definite and effective method of treatment. However, early detection of the tumor play an important role, MRI is now the most important method for the detection of tumor extent, which facilitates the treatment choice as well as the prediction of prognosis. In our case, we followed-up the patient five years postoperatively by MRI and got a good result.

## Background

Desmoid tumor, also known as aggressive fibromatosis, is histologically benign fibrous lesion with aggressive infiltration of adjacent tissues and high local recurrence rate but without metastatic potential. It is a relatively rare neoplasm accounting for 3% of soft tissue tumors and 0.03% of all neoplasms. Main locations of desmoid tumor occurrence are the abdominal wall, proximal extremities, and the mesenteric intestine in patients with familial adenomatous polyposis (FAP) [1]. Desmoid tumor covers a broad spectrum of fibromatoses, which arise from the connective tissue, fascia or aponeurosis of a muscle [2]. The exact pathogenesis of desmoid tumor is unknown, however, genetic abnormalities (Familial adenomatous polyposis and Gardner’s syndrome), sex hormones and trauma have been considered as causative factors [1]. In our case the patient reported to have undergone extensive walking before the tumor occurred. This leads to the assumption, that the growth of the tumor could have been induced by this repeated minor trauma to the ligament, as is reported by Stollwerck et al. [3]. The incidence of desmoid tumor is reported to be 2.4 to 4.3 cases/1 million/year, whilst the majority of desmoid tumors are found intraabdominally and only 7–15% of which are found in the extra-abdominal area.

## Case presentation

A 49-year-old man presented with a 2-month history of posteromedial knee dull pain and decreased range of motion of the knee. The patient also complained of subjective swelling of the invovled lower leg. The general physical examination was unremarkable. Examination of the knee showed no effusion, there was no palpable inguinal or popliteal lymphadenopathy, neither warmth nor erythema was detected. There was no ligamentous instability or joint line tenderness. The only and most significant positive sign was that the range of motion of the right knee was limited to 0°-90°. The rest of his musculoskeletal examination was normal. The laboratory workup was unremarkable. No recent trauma or major injury had occurred, but the patient walked 4–5 hours per day to work for about 6 months, which might have some business with it according to the patient himself.

**Figure 1:**
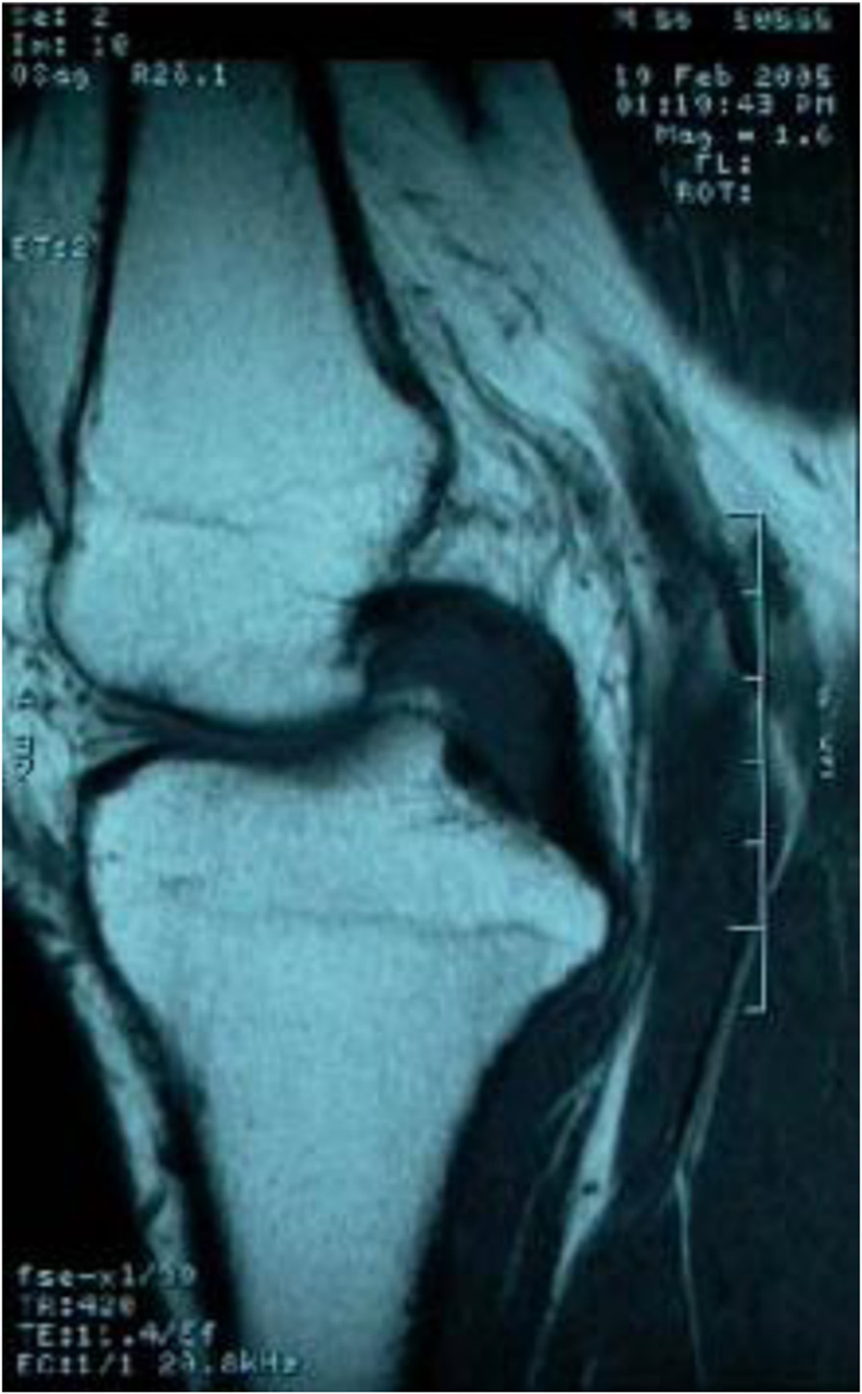
Sagittal, coronal and axial T1-weighted MR image showed slight hypo-intensity lesion similar to muscle and peripheral hypo-intensity ring.

**Figure 2:**
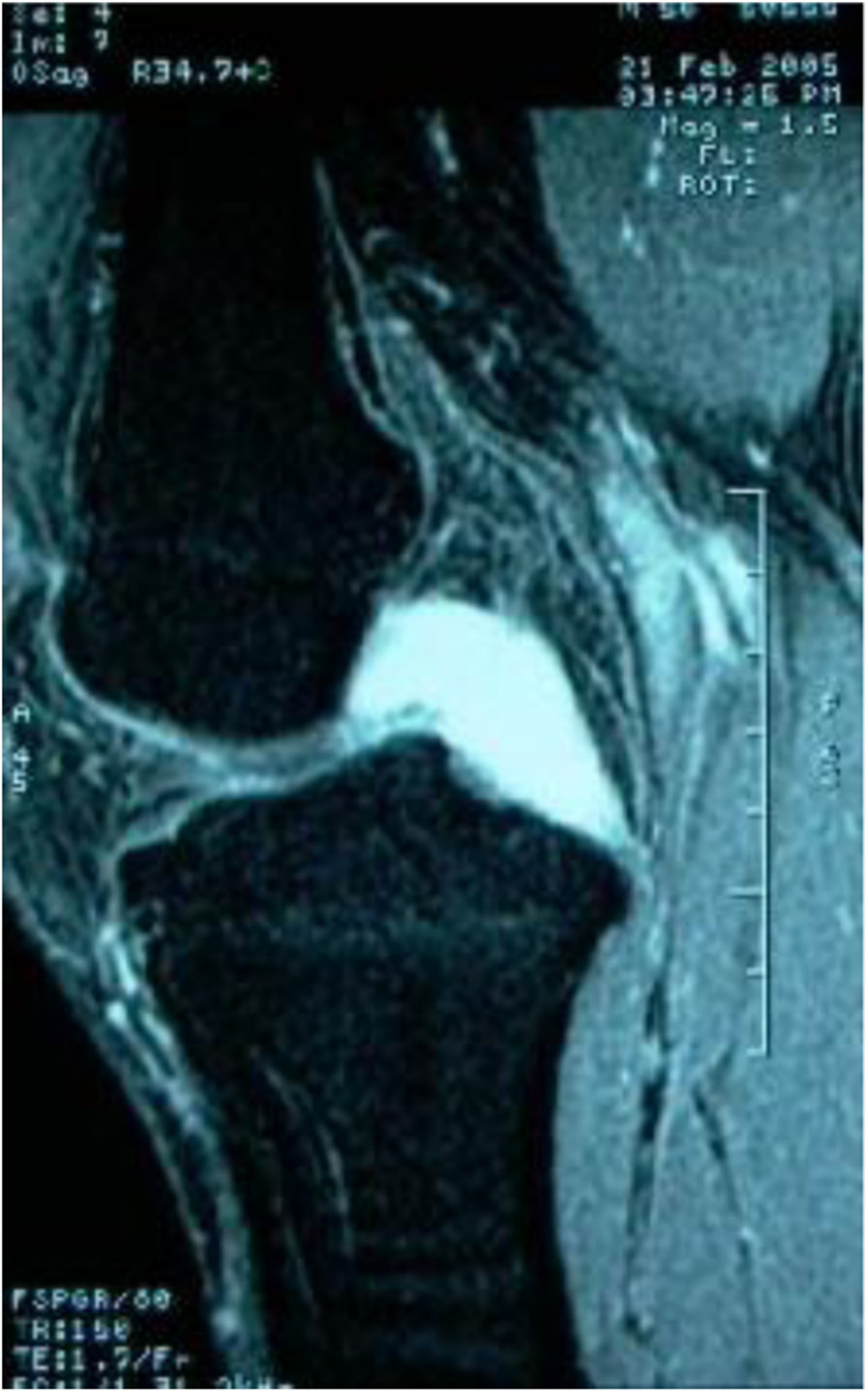
Sagittal fat-suppressed T2-weighted MR image showed hyper-intensity lesion with distinct border localized in posterior cruciate ligament.

**Figure 3:**
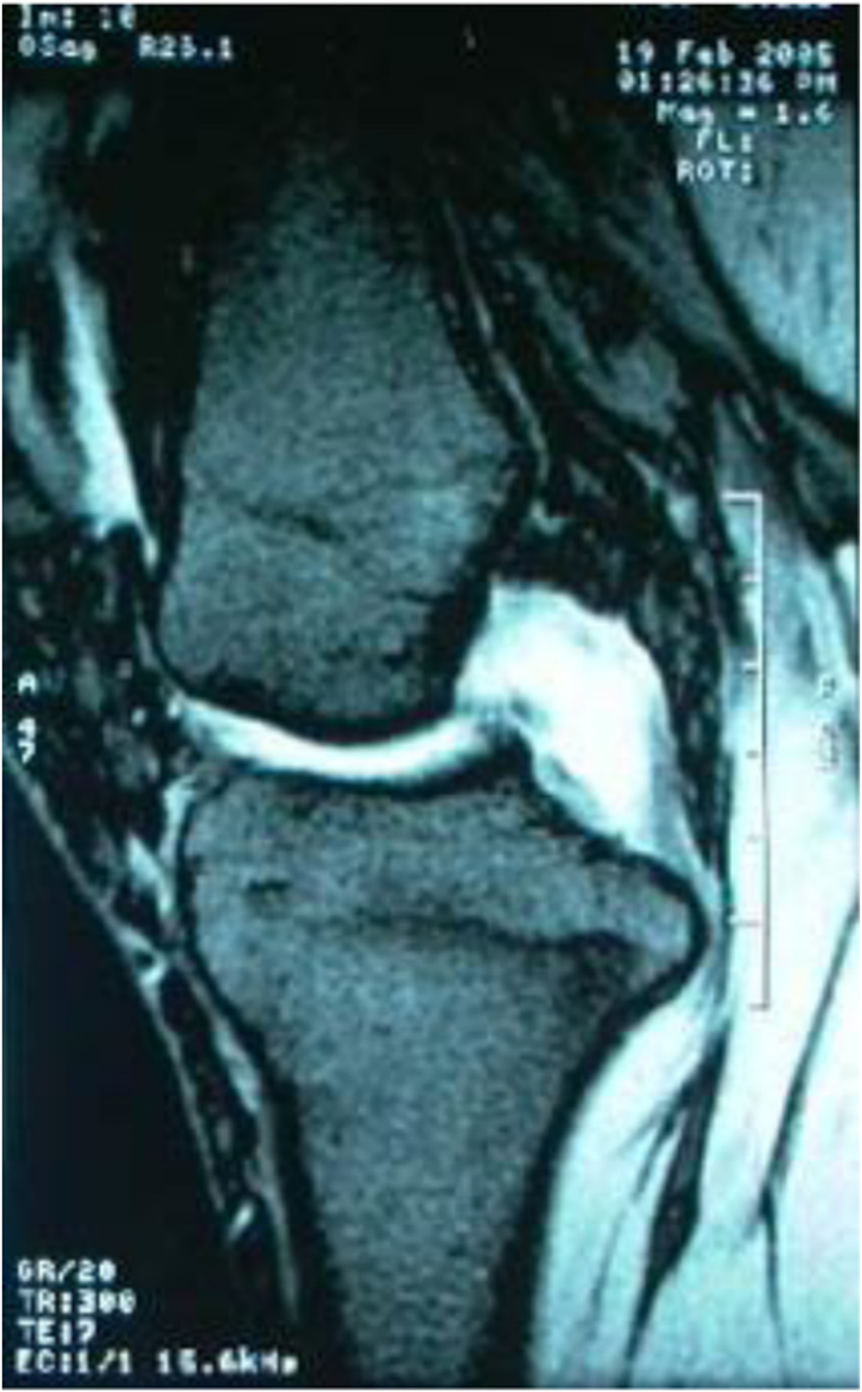
Contrast MR image showed slightly heterogeneous moderate enhancement and hypo-intensity ringlike non-enhancement.

Plain radiographs of the right knee were unremarkable except for mild degenerative changes. Computed tomographic (CT) examination was not carried out because of financial concern. T1-weighted MRI (Figure 1) showed slight hypo-intensity lesion similar to muscle and peripheral hypo-intensity ring. Fat-suppressed T2-weighted MRI (Figure 2) showed hyper-intensity lesion with distinct border localized in posterior cruciate ligament. Contrast MR image (Figure 3) showed slightly heterogeneous moderate enhancement and hypo-intensity ringlike non-enhancement.

**Figure 4:**
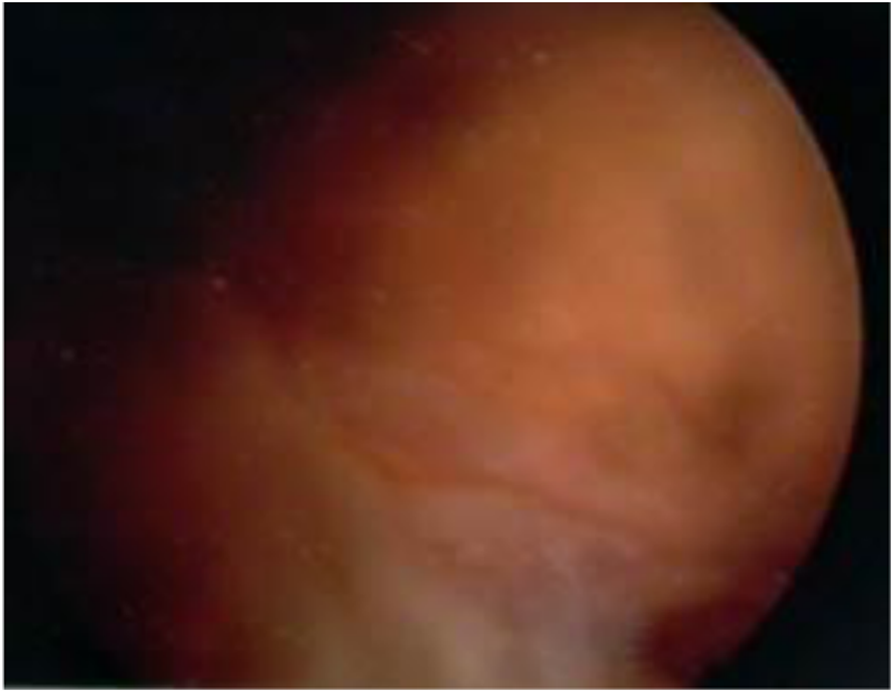
The PCL appeared wider and grayer than normal on gross examination, and it was tough and inelastic.

**Figure 5:**
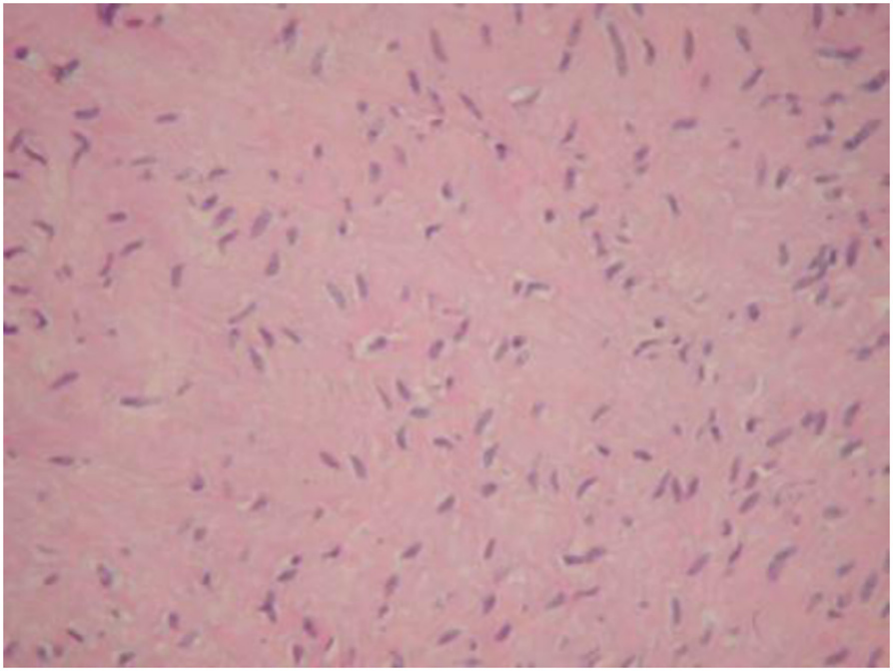
Histopathological evaluation revealed uniform proliferation of spindle cells, with a moderate amount of collagen fibers, suggesting desmoid tumor.

Firstly, diagnostic arthroscopy was performed. The posterior cruciate ligament appeared wider and grayer than normal on gross examination, and it was tough and inelastic (Figure 4). Intraoperative frozen histology showed a uniform proliferation of spindle cells with a moderate amount of collagen fibers led to a diagnosis of desmoid tumor in the PCL (Figure 5). Then the PCL was completely resected by clipper bit by bit, and the remaining part of the starting and ending points of the tendon were cleared off by plane iron and low temperature radiofrequency ablation. After that it was reconstructed by Four-strand semitendinosus and gracilis tendon autograft arthroscopically directly after the intraoperative histology and resection. Endobuttons were used to fix the ends of the transplanted ligment. No adjuvant treatment was given postoperatively.

**Figure 6:**
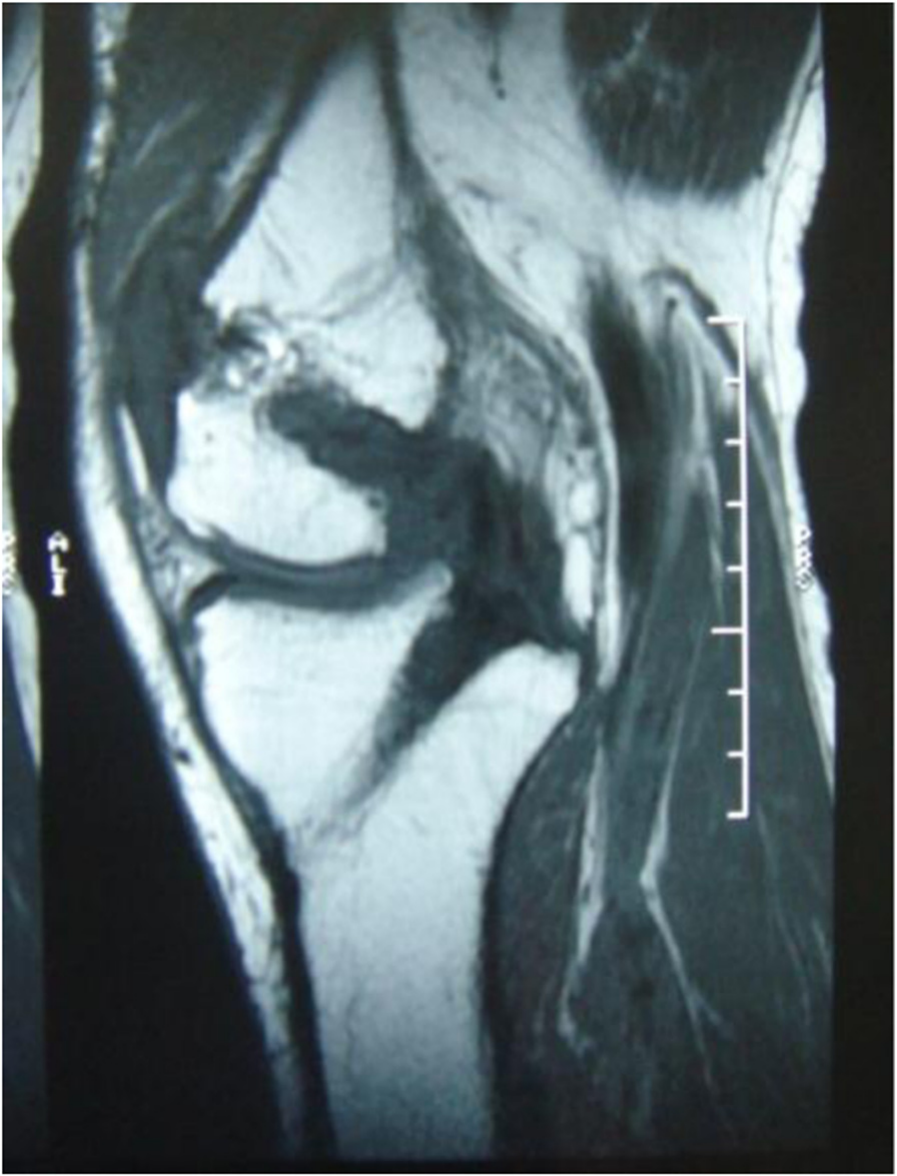
MRI of left knee 5 years after operation showing the satisfactory morphology of the reconstructed PCL and no signs of recurrence.

Since then, the patient has remained asymptomatic without any pain around the knee, and after a six-month rehabilitation process, the range of motion of the involved knee reached 0°-130°. Now five years after operation, there were no clues as to recurrence of the tumor examined by MRI (Figure 6).

## Discussion

Desmoid tumor is characterized by infiltrative growth and a tendency towards recurrence; however, unlike sarcoma, it never metastasizes. To avoid local recurrence, an early diagnosis is required for this type of tumor, which is difficult as most of these patients are asymptomatic like other kinds of rare tumors within the knee including Intra-articular ganglia, Extra-osseous osteochondroma, pigmented villonodular synovitis and even sarcoma inside the knee [4-8]. At the same time, it is also important to preoperatively detect the exact localization and extent of the lesion in this tumor, and MRI is now the most important method for detection of the tumor, diagnosis, decision making and follow-up. In our case, MRI showed that the pathological changes were limited within PCL, after a complete resection and one stage functional reconstruction, we followed-up the patient five years postoperatively by MRI and there were no clues as to recurrence of the tumor. Here, we think the early stage diagnosis and complete resection contribute mainly to the good results.

Desmoid tumor of the abdominal wall mostly occurs in women during pregnancy or soon after delivery, whereas extra-abdominal desmoid tumor is more frequent from puberty to 50 years of age, with both men and women equally affected. Extra abdominally desmoid tumor may occur in a variety of anatomical locations, including the muscles of the shoulder, the chest wall and back, thigh, and head and neck. However, solitary occurrence in PCL of the knee has never being reported. Here, we present a case of desmoid tumor in the cruciate ligament of knee joint. Resection and functional reconstruction was successfully performed, and the patient has been tumor-free for five years after surgery.

Unexplained etiology and the various locations make the treatment of desmoid tumor extremely difficult. As this tumor entity is rare, data giving evidence based recommendations for the optimal treatment algorithm for this disease is lacking [9]. At present optimal treatment of desmoids remains controversial, and there is no definite and effective method of treatment. However, complete resection or a wide surgical excision is the first choice of therapy for this type of tumors, as was advocated nearly 100 years ago [1], although Some authors affirmed that there were no significant difference between negative margins and microscopic positive margin regarding recurrence rate [10-12]. For patients with desmoid tumors that are not amenable to surgery, radiotherapy or adjuvant therapy using non steroidal anti-inflammatory agents, tamoxifen, interferon, anti-neoplastic agents, either alone or in combination have been reported to be useful in some cases, but unfortunately, the exact benefit offered by them is lack of evidence due to non-sufficient patient numbers and missing prospective randomized studies, although The French sarcoma group recently reported encouraging results in patient treated with chemotherapy [13]. We did not apply any above adjunctive treatment of choice and fortunately, the patient has been tumor-free for five years after surgery. For this patient we think early detection of the tumor because of the proper application of MRI in the beginning and complete resection is the key role of the good results, but still longer term curative effect remains to be followed-up.

## Conclusion

Early detection of desmoid tumor and complete resection play an important role in its prognosis, and MRI is a good choice for its early detection.

## Consent

Written informed consent was obtained from the patient for publication of this case report and any accompanying images.

## Competing interests

The authors declare that they have no competing interests. No financial support has been received.

## Authors’ contributions

WL and SK have been involved in drafting the manuscript and revising it critically for important intellectual content; ZW, LD have made substantial contributions to conception and design, acquisition of data, analysis and interpretation of data; WH has taken part in the whole procedure. All authors read and approved the final manuscript.

## Pre-publication history

The pre-publication history for this paper can be accessed here:

http://www.biomedcentral.com/1471-2474/14/69/prepub
